# Therapeutic effects of stem cells from human exfoliated deciduous teeth on diabetic peripheral neuropathy

**DOI:** 10.1186/s13098-019-0433-y

**Published:** 2019-05-17

**Authors:** Jing Xie, Nanquan Rao, Yue Zhai, Jingzhi Li, Yuming Zhao, Lihong Ge, Yuanyuan Wang

**Affiliations:** 1 Department of Pediatric Dentistry, School and Hospital of Stomatology, Peking University, #22 Zhongguancun Nandajie, Haidian District, Beijing, 100081 China; 2 Department of Stomatology, Shenzhen Children’s Hospital, #7019, Yitian Road, Shenzhen, 518026 China

**Keywords:** Stem cells from human exfoliated deciduous teeth, Diabetic peripheral neuropathy, Goto-Kakizaki rats, Stem cell therapy

## Abstract

**Objective:**

To evaluate the therapeutic potential of stem cells from human exfoliated deciduous teeth (SHED) for diabetic peripheral neuropathy.

**Methods:**

The biological characteristics of SHED were identified by flow cytometric study and evaluation of differentiation potential. Using high-fat feeding, diabetes was induced in GK rats, and SHED were transplanted into the caudal veins of these rats. Immunohistochemical analysis was used to compare the capillary to muscle fiber ratio and intra-epidermal nerve fiber density between SHED- and saline-treated diabetic rats. Further, the expressions of angiogenesis-related and neurotrophic factors were quantified by real-time PCR and western blot.

**Results:**

SHED had a capacity of multiple differentiation and shared typical characteristics of mesenchymal stem cells. SHED transplantation relieved diabetic neuropathic pain, enabled functional recovery of the peripheral nerves, and increased the capillary to muscle fiber ratio and intra-epidermal nerve fiber density compared to the saline group and normal controls. Real-time PCR results showed that the expressions of CD31, vWF, bFGF, NGF, and NT-3 in the skeletal muscles were higher in the SHED group than in the saline groups. Western blot results indicated that the levels of the CD31 and NGF proteins were higher in the SHED transplantation group than the saline group.

**Conclusion:**

SHED transplantation ameliorated diabetic peripheral neuropathy in diabetic GK rats. Thus, systemic application of SHED could be a novel strategy for the treatment of diabetic peripheral neuropathy.

## Background

Diabetes has become a highly prevalent metabolic disorder worldwide. According to statistics released by the International Diabetes Federation, about 8.8% of 20- to 79-year-old adults had diabetes in 2017, an equivalent of 425 million people [1].

Diabetic peripheral neuropathy (DPN) is a common complication in both type 1 and type 2 diabetes. The abnormal peripheral sensations experienced by patients with this condition include paresthesia, allodynia, hyperalgesia, and spontaneous pain. The pain is characterized by superficial skin pain, like cutting or burning and numbness to temperature. Sometimes, patients experience less sweating, dry skin, vasoconstriction disorders, and other manifestations of autonomic neuropathy. However, tendon reflexes and muscle movements are usually normal. DPN often occurs in the lower limbs and feet, and many patients experience a marked reduction in quality of life [2].

Various pathogenic factors are involved in the onset and progress of DPN, including microvascular lesions [3–5]; changes in the osmotic pressure; and the formation of glycosylation terminal product caused by the metabolic disorder, oxidative stress response [6], and the lack of neurotrophic factors such as nerve growth factor [7]. Pathologic changes like progressive distal axonal degeneration, axonal loss, and demyelination accompanied by microvascular changes could be observed under such circumstances.

Pharmacological treatment with neurotrophic drugs, microcirculation improvement drugs, and antioxidant drugs is effective for DPN, but the effects are partial in many cases [8–10]. Relieving the symptoms of DPN, therefore, remains an important issue for many clinicians, and more effective treatment protocols are needed.

With the development of stem cell research, many studies about the use of stem cells for treatment of diabetes and its complications have achieved good results. Endothelial progenitor cells [11] and induced pluripotent stem cells [12], bone marrow mononuclear cells [13], mesenchymal stem cells [14, 15], including dental pulp stem cells [16], can reduce hyperalgesia in diabetic rats and increase motor and sensory nerve conduction velocity, sciatic nerve blood flow, and capillary and nerve fiber densities.

Stem cells from human exfoliated deciduous teeth (SHEDs) have been identified as a novel population of mesenchymal stem cells (MSCs) capable of differentiating into a variety of cell types including neural cells, odontogenic cells, and adipocytes [17]. Systemic SHED transplantation showed effective improvements in immune disorders [18]. Importantly, SHEDs are derived from a readily accessible tissue source, namely, human deciduous teeth that are expendable and routinely exfoliated in childhood with little or no morbidity.

In the present study, we test our hypothesis that SHED transplantation has therapeutic potential in DPN.

## Methods

### Ethics statement

This study was carried out in strict accordance with the recommendations in the Guide for the Care and Use of Laboratory Animals of the Stomatological Hospital of Peking University. All efforts were made to minimize the animals’ suffering. This study was performed under an ethics protocol previously approved by Ethics Committee of the Peking University Health Science Center (Approval No: LA2018231).

### Isolation and characterization of SHEDs

#### Isolation of SHEDs

Retained deciduous teeth were extracted from 6- to 10-year-old children (15 patients) under local anesthetic at the Stomatological Hospital of Peking University. Informed consent was obtained from the patients before this.

The deciduous teeth were repeatedly washed using phosphate-buffered saline (PBS; 0.01 M, pH = 7.4). Dental pulp was extracted using a barbed nerve broach, washed twice with sterile PBS supplemented with antibiotics (100 U/mL penicillin and 100 g/mL streptomycin), and mixed with 3 mg/mL type I collagenase and 4 mg/mL dispase at a ratio of 1:1, placed in a water bath at 37 °C for 1 h, digested, and finally centrifuged at 1000 rpm/min for 5 min. The supernatant was discarded, the SHED pellet was re-suspended in an appropriate volume of culture solution, and passed through a filter of pore size 70 μm. The cell suspension was inoculated in a 25 cm^2^ flask, supplemented with penicillin–streptomycin solution and α-MEM solution containing 15% fetal bovine serum, and cultured at 37 °C in 5% CO_2_ for 3 days. Half the culture solution was changed every 3 or 4 days. When monolayer cell confluence was observed, the cells were passaged at a ratio of 1:3.

#### Stem cells markers profile

The surface marker profiles of SHED were tested with flow cytometry. The fourth passage of cells were resuspended in cold PBS containing 2% FBS at a concentration of 1 × 10^6^ cells/mL prior to adding the following antibodies: CD73 (Brilliant Violet421, Biolegend), CD90 (FITC, BD Pharmingen), CD105 (PE, Biolegend) and CD45 (PerCP/Cy5.5, Biolegend). All experiments included negative controls without antibodies or with corresponding isotype controls. The flow cytometer was set using isotype controls. Cells were gated by forward and sideward scatter to eliminate debris. Then the stained cells were analyzed with a Beckman Coulter flow cytometry system (FC500, Beckman Coulter, Brea, CA, USA).

#### Differentiation of SHED into multiple lineages

To induce odonto/osteogenic differentiation, cells were incubated in α-MEM containing 10 mM β-glycerophosphate, 50 mg/mL ascorbate phosphate, 10 nM 1,25-dihydroxy vitamin D3, 10 nM dexamethasone, and 15% FBS for 3 week. And the mineralization was detected by staining with 1% Alizarin Red S.

To induce adipogenic differentiation, cells were incubated in adipogenic medium containing 0.5 mM 3-isobutyl-1-methylxanthine, 100 μM indomethacin, 1 mg/mL insulin, 1 mM dexamethasone, and 15% FBS for 4 week. Lipid droplets were stained with 2% (w = v) Oil Red O reagent.

To induce chondrogenic differentiation, ‘‘pellet culture’’ technique was used. Briefly stated, approximately 250,000 cells were placed in a 15 mL polypropylene tube (Falcon), and centrifuged to pellet. 0.5 mL of chondrogenic medium (PT-3003; Cambrex Bio Science, Verviers, Belgium) was added, freshly supplemented with 10 ng/mL of transforming growth factor-b 3 (TGF-b 3). After 4 weeks, the pellets were fixed, embedded in paraffin, and cut into 5-mm sections. Then the sections were evaluated via immunohistochemistry for the expression of collagen type II (Col II; CIIC1; Developmental Studies Hybridoma Bank).

### Animal model establishment

Ten-week-old male specific pathogen-free Goto-Kakizaki (GK) rats weighing about 250–300 g were purchased from Changzhou Cavens Laboratory Animal, Inc. (Changzhou, China). The rats were maintained on a 12 h light:12 h darkness cycle with free access to rodent chow and water. After they consumed a high-fat diet, their blood glucose levels were determined; rats that had blood glucose levels over 11.1 mM for 3 consecutive days were classified as diabetic. Diabetic rats were then fed conventional chow for an additional 8 weeks, and their blood glucose levels were determined again. When the rats experienced mechanical hyperalgesia and any one random diabetic rat showed pathologic changes, establishment of a DPN animal model was confirmed. Then, 1 × 10^7^ green fluorescent protein (GFP)-transfected SHEDs were implanted in the caudal veins of two DPN GK rats in order to observe the location of transplanted SHEDs in the skeletal muscles. A total of 20 GK rats with diabetic nephropathy were divided into two groups, according to a random number table (n = 10 in each group). The SHED treatment group received 1 × 10^7^ cells implanted in the caudal vein, given in two shots administered 2 weeks apart. The SHEDs were re-suspended with 1.0 mL saline. The saline group received 1 mL saline injected into the caudal vein twice. Ten male specific pathogen-free Wistar rats of similar age and body weight as the experimental animals (250–300 g) were used as normal controls.

### Measurement of mechanical hyperalgesia

All tests were performed in a blinded fashion. Baseline test was carried out before SHED infusion. Additionally, the same tests were repeated at 1 weekly intervals for 12 weeks. Each rat was individually placed in an inverted transparent cage with a wire mesh bottom and was habituated to the test chamber for at least 30 min in advance before the test. According to Dixon’s up and down method [19], pain-related behavior induced by mechanical stimulation was measured with von Frey hairs (VFH; North Coast Medical, USA). Each VFH was repeated perpendicularly 6 times (once every 2–3 s) to the unilateral mid-plantar hind paw, the foot withdrawal behavior of each rat was recorded. The paw withdrawal mechanical threshold (PWMT) was then calculated as (10^[Xf+kδ]^)/10000. A significant decrease in PWMT was interpreted as mechanical hyperalgesia.

### Tissue collection

After the last behavioral test, rats were killed using an overdose of pentobarbital, and the soleus muscles, footpads, and sciatic nerves were obtained. The harvested tissues were immediately frozen in liquid nitrogen and stored at − 80 °C for molecular biological studies, while the other was stored in 4% paraformaldehyde solution at 4 °C overnight and embedded in paraffin for histological analysis.

### Weil staining for histological examination

The paraffin-embedded sciatic nerve was sliced into 10-μm sections. After 10 min of staining with hematoxylin, the sections were washed twice in distilled water, differentiated with alum solution, washed twice, treated with Weil staining reagents, and dehydrated in gradient ethanol and dimethylbenzene for 15 min.

### Capillary to muscle fiber ratio

The soleus muscles samples were cut into 5-μm sections. The slides were deparaffinized and rehydrated subsequently. Primary CD31 antibody (anti-PECAM-1 polyclonal antibody; Abcam, USA) diluted 1:200 were incubated on slides overnight at 4 °C. And the secondary antibody used for visualization was Alexa Fluor 488 anti-rabbit antibody (Abcam, USA). The sections were stained with 4′-6-diamidino-2-phenylindole (DAPI) and mounted, and then observed under fluorenscence microscopy and images were captured with a digital camera. According to the methods described by Hata^16^, the number of capillary and muscle fibers was counted blindly in 10 fields from each section by three independent investigators and then the ratios (capillary to muscle fiber) were calculated out.

### Intra-epidermal nerve fiber density

The footpads samples were cut into 8-μm sections. The deparaffinized and rehydrated sections were incubated with the primary anti-PGP9.5 antibody (Zhongshanjinqiao, China) diluted 1:400 overnight at 4 °C. And then a DAB (3′-3-diaminobenzidine) kit (Zhongshanjinqiao, China) was used for staining. The individual nerve fiber in the epidermis from the dermis in 10 fields from each section was measured blindly by three investigators. Intra-epidermal nerve fiber density (IENFD) and the number of epidermal nerve fibers per length of the epidermal basement membrane were recorded and calculated.

### Relative mRNA expression in hind limb skeletal muscles 

RNA extraction from the hind limb skeletal muscles was performed using the TRIzol reagent (Invitrogen, USA) according to the manufacturer’s instructions. The cDNA was synthesized using HiFi-MMLV cDNA (CWbio Co Ltd., China). cDNA was amplified by PCR using the following primers(forward primer,reverse primer): VEGF: 5′-CCAGGCTGCACCCACGACAG-3′, 5′-TCATTGCAGCAGCCCGCAC-3′; CD31: 5′-ACCGTCCAGAAGAACTCCAATGA-3′, 5′-ACAGAGCACCGAAGCACCAT-3′; vWF: 5′-CTCCAGCCACATTCCATACAATCT-3′, 5′-CCTCCAATCAACACAGACTCCATT-3′; bFGF: 5′-AGAGGAGTTGTGTCCATCAAG-3′, 5′-CTCCAGGCGTTCAAAGAAGA-3; NGF: 5′-TGCATAGCGTAATGTCCATGTTG-3′, 5′-TGTGTCAAGGGAATGCTGAAGT-3′; NT-3: 5′-GACACAGAACTACTACGGCAACAG-3′, 5′-CTCCAGGCGTTCAAAGAAGA-3′; GAPDH: 5′-TGGAGTCTACTGGCGTCTT-3′, 5′-TGTCATATTTCTCGTGGTTCA-3′.Real-time quantitative PCR was performed with the Line Gene 9600 Plus System (Bioer, China) and the relative value of the gene expression was calculated by the ΔΔCt method.

### Protein expression in hind limb skeletal muscles

Using an ultrasonic processor, the tissues were separated after being washed twice with PBS. They were collected in a 1.5 mL tube, and radio immunoprecipitation assay buffer was added to obtain lysate. The protein sample was quantified using the BCA protein assay, and western blot analysis was used to measure the expression levels of the CD31 and NGF proteins. A Tanon-4100 chemiluminescence analyzer (Biotanon Shanghai, China) was used for detection and photographing. The integral optical density value of the target protein was calculated and statistically compared with that of β-actin. The result was verified using an enhanced chemiluminescence system (Pierce, US).

### Statistical analysis

SPSS statistical software was used for data analysis (SPSS Inc., USA). All data were expressed as mean ± standard deviation (SD). ANOVA was used for multi-group comparison. A *P* value of less than 0.05 was considered as statistically significant.

## Results

### Characterization of SHED

**Fig. 1:**
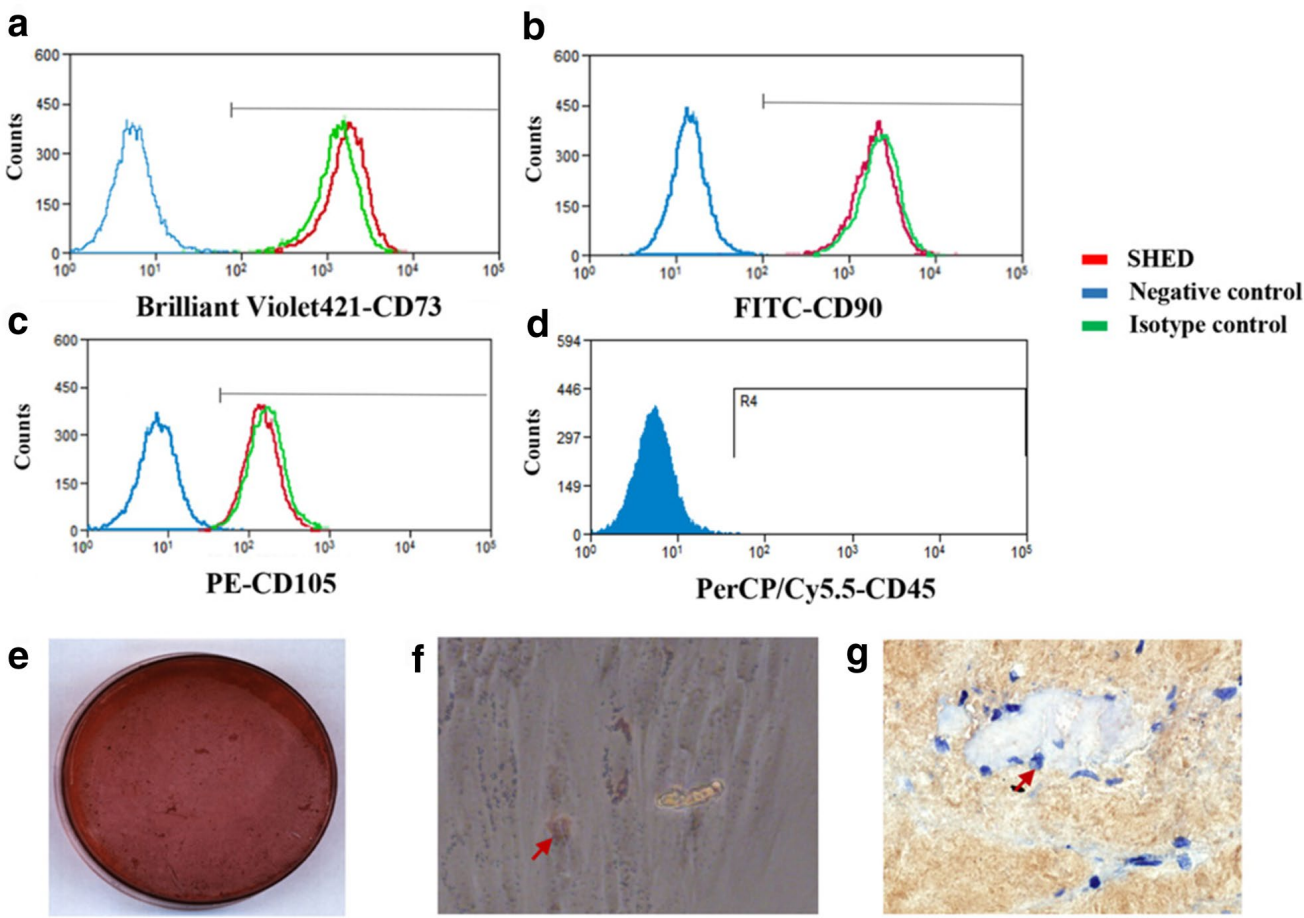
Isolation and biological characterization of SHED. Flow cytometry analysis revealed that SHED express the MSC markers CD73 (**a**), CD90 (**b**) and CD105 (**c**), while lack expression in blood cell marker CD45 (**d**). SHED have the ability of multilineages of differentiation. They could be induced to form mineralized nodules, lipid droplets and chondrocytes, as indicated by arrows in (**e**–**g**), respectively. Developmental potential of SHED ex vivo

Flow cytometric results indicated SHED showed a positive expression of MSC markers CD73, CD90, and CD105, meanwhile a lack expression of CD45 which is a marker of hematopoietic cells (Fig. 1a–d). in addition, SHED had the ability to differentiate into multiple lineages. After induction, condensed nodules positive for alizarin red S (Fig. 1e), oil red O positive lipid droplets (Fig. 1f) and collagen II positive chondrocytes (Fig. 1g) were observed.

### Effects of SHEDs on mechanical hyperalgesia

**Fig. 2:**
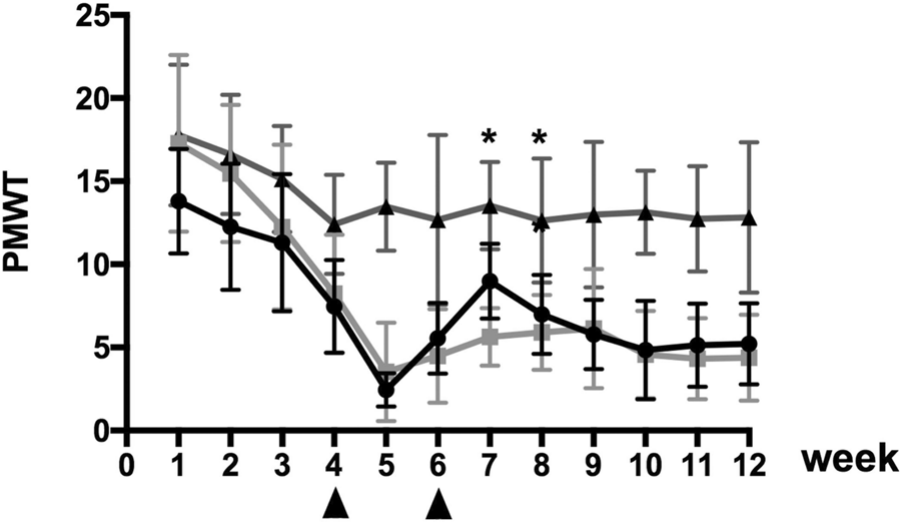
Effects of SHED transplantation on mechanical hyperalgesia in the hind paws of rats. After SHED transplantation for 6 weeks, the PWMT increased significantly and remained high for 2 weeks (*P < 0.05)

The effects of SHED transplantation in the caudal vein on mechanical hyperalgesia in the hind paws of diabetic rats were shown (Fig. 2). The PWMT decreased significantly with the increase in the blood glucose concentration. After SHED transplantation for 6 weeks, the PWMT increased significantly and remained high for 2 weeks. These results indicate that SHED transplantation in the caudal vein inhibited mechanical hyperalgesia in diabetic rats.

### Localization of transplanted SHEDs

**Fig. 3:**
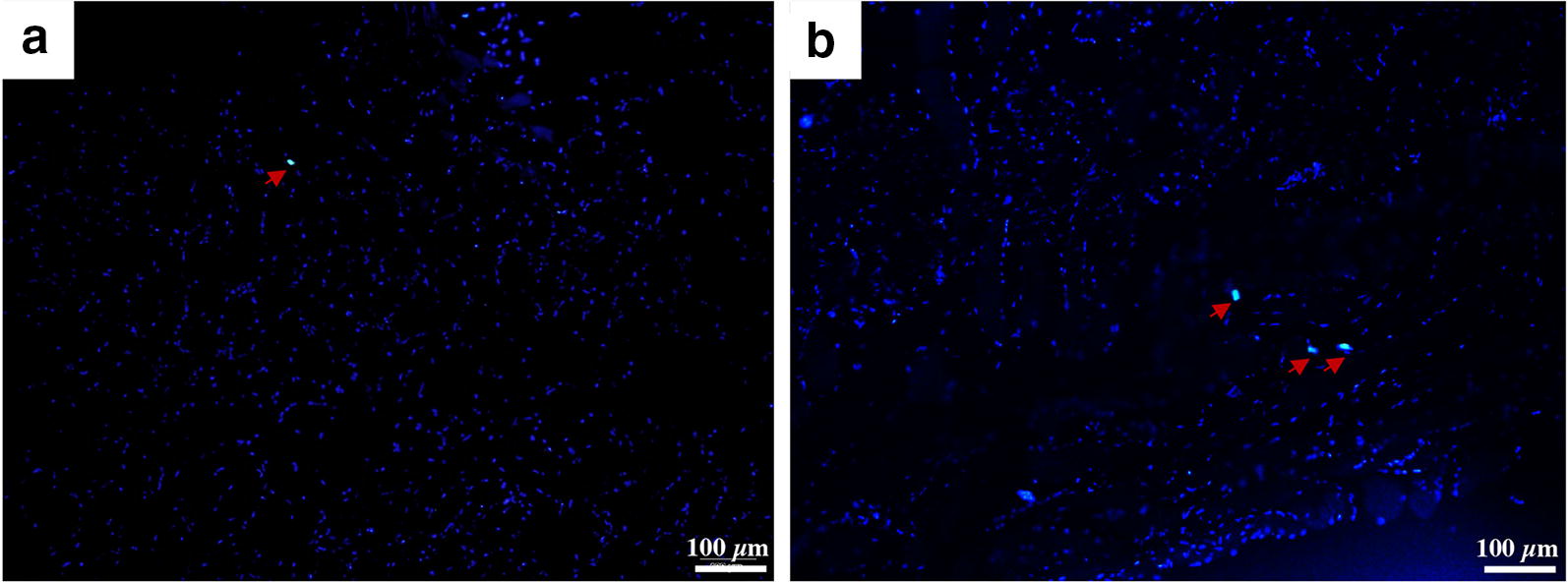
Localization of transplanted SHEDs. The transplanted GFP-SHEDs (Red arrowheads) were localized around the muscle bundles in the skeletal muscles (**a**, **b**). Bar = 100 μm

Our findings showed that the transplanted GFP-SHEDs were localized around the muscle bundles in the skeletal muscles (Fig. 3). The GFP-SHEDs were labelled in red arrow.

### Histological observations

Weil staining showed that the sciatic nerve fibers in the control group were closely arranged and uniformly distributed. The color and thickness of the myelin sheath were uniform. The sciatic nerve fibers in the saline group were loosely arranged, and demyelination was detected.

**Fig. 4:**
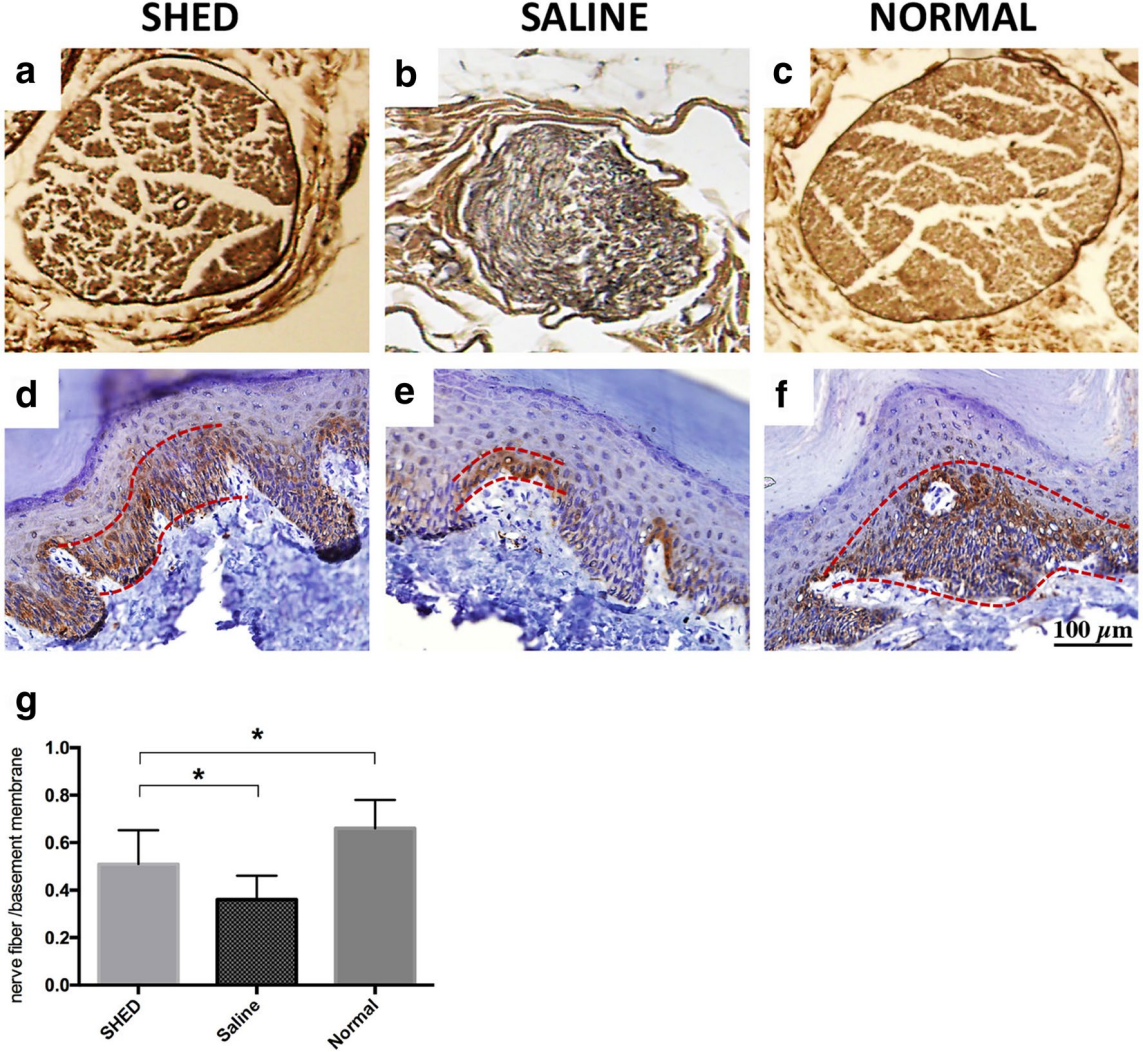
Histological observation of the sciatic nerves of rats. SHED (**a**) and groups (**b**) and normal controls (Weil staining; ×4). The nerve fibers in the control group are closely arranged and uniformly distributed (**c**). The color and thickness of the myelin sheath are uniform. Those in the saline group are loosely arranged and demyelination can be seen. The characteristics of the sciatic nerve fibers in the SHED group are between those of the other two groups. PGP 9.5 immunostaining for examination of IENFD in the footpads. SHED (**d**) and saline groups (**e**) and the normal controls (**f**). SHED transplantation significantly improved the IENFD to levels better than those in the saline group (**g**) (*P < 0.05). Bar = 100 μm, all images were taken under the same magnification

The pathological features of the sciatic nerve fibers in the SHED group were somewhere between those of the other two groups. After 4 weeks of stem cell transplantation, the sciatic neuropathology in the rats improved significantly (Fig. 4a–c).

To determine the extent of damage to the sensory small nerve fibers in DPN, we analyzed the IENFD at the footpads. Intra-epidermal nerve fibers were visualized using PGP9.5 immunostaining. Quantitative analyses showed that the IENFD was significantly lower in diabetic rats than in normal rats. IENFD significantly improved with SHED transplantation to levels higher than those in the saline group (Fig. 4d–g).

### SHED transplantation increased the capillary density of skeletal muscles in diabetic rats 

**Fig. 5:**
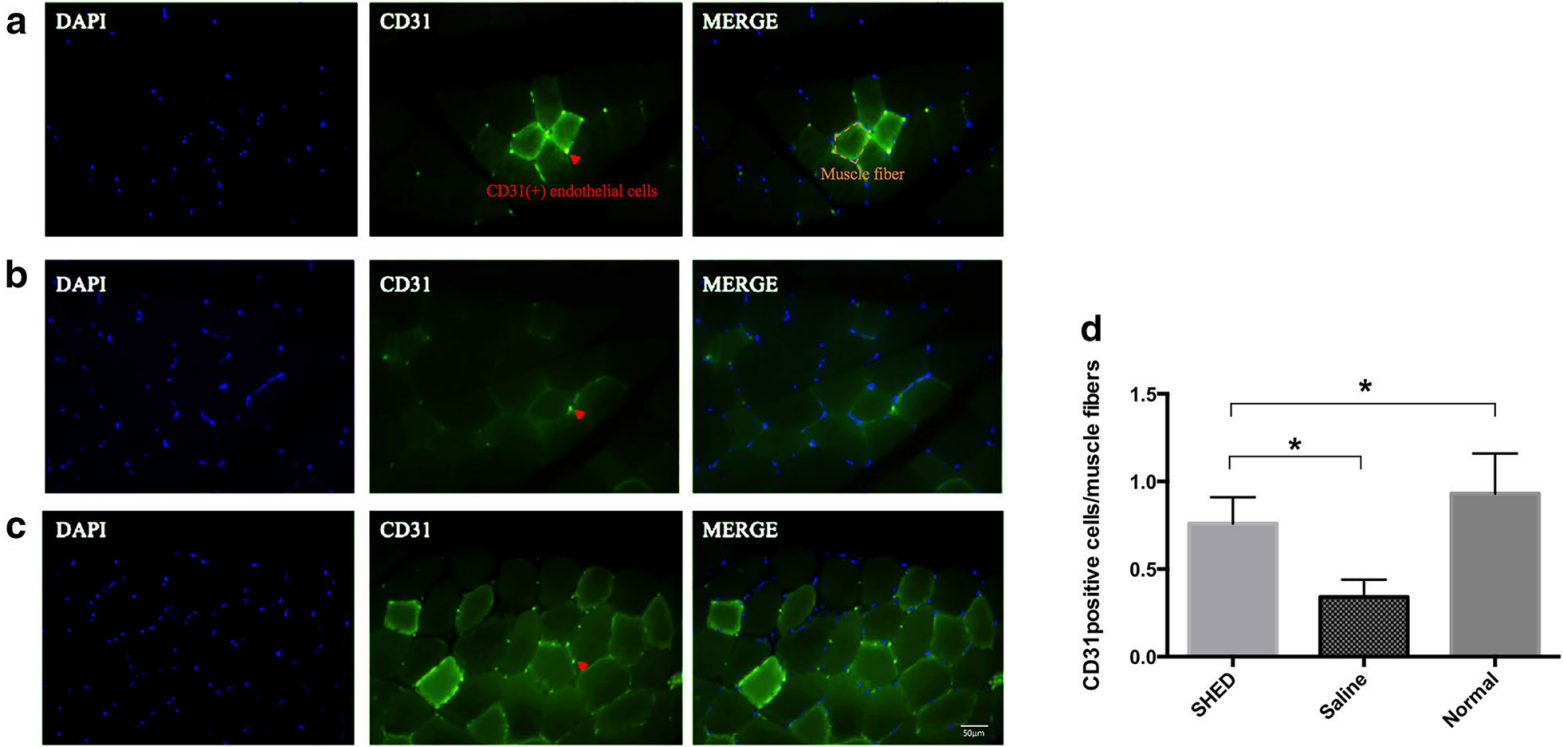
Capillary to muscle fiber ratio. SHED (**a**) and saline groups (**b**) and the normal controls (**c**). SHED transplantation significantly increased the ratio of CD31-positive cells (capillaries) to muscle fibers in the diabetic rats (**d**) (*P < 0.05)

The capillaries in the soleus muscles were visualized using CD31 immunofluorescence staining. Quantitative analyses revealed that the ratio of CD31-positive endothelial cells to muscle fibers was significantly lower in the diabetic rats than in the normal control rats. SHED transplantation significantly increased (P < 0.05) this ratio in diabetic rats (Fig. 5).

### Rt-PCR

**Fig. 6:**
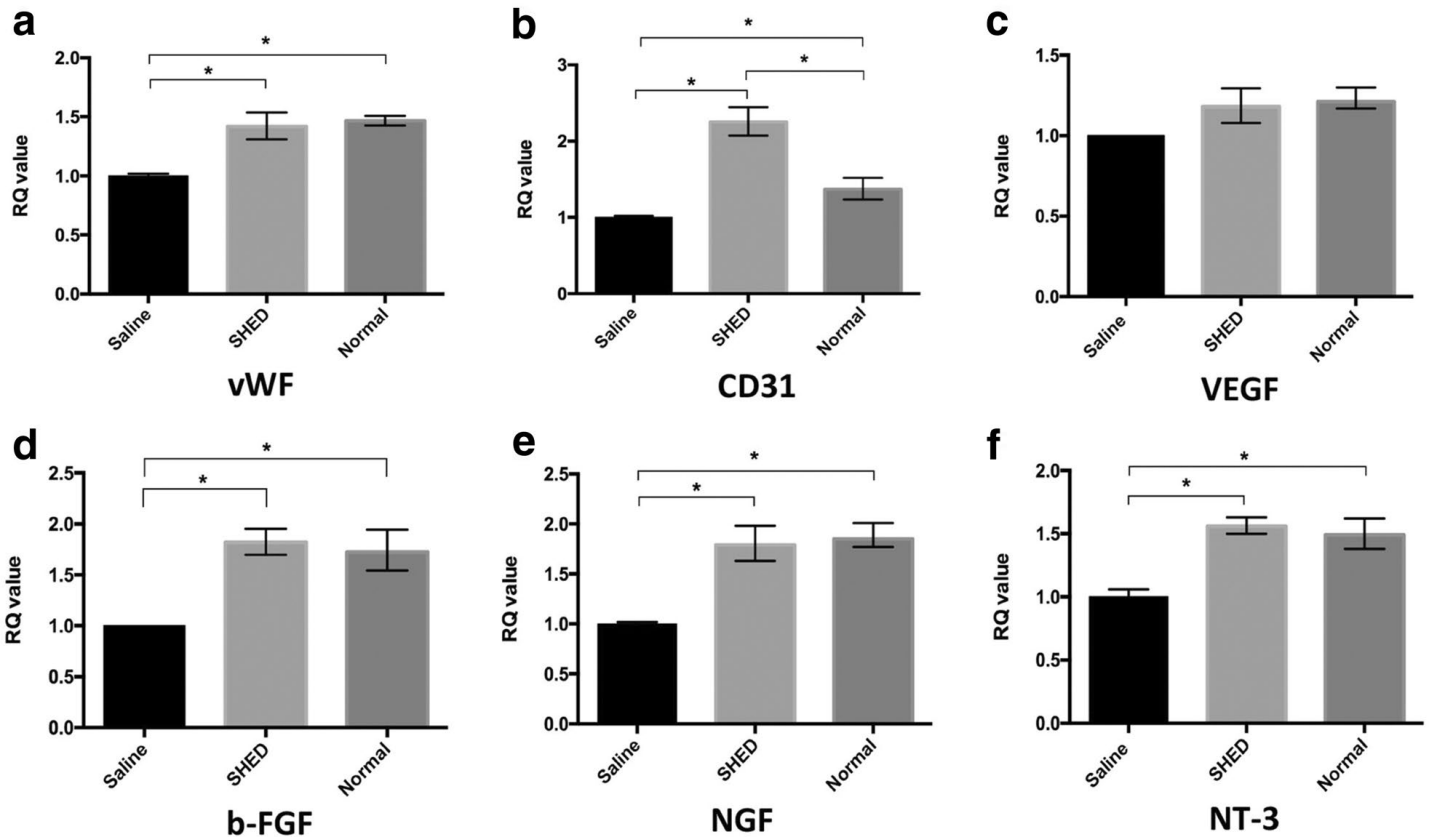
Real-time quantitative PCR. vWF (**a**), CD31 (**b**), VEGF (**c**), b-FGF (**d**), NGF (**e**), and NT-3 (**f**) mRNA were examined. The expressions of vWF, CD31, b-FGF, NGF, and NT-3 mRNA in the SHED group were higher than those in the saline group (*P < 0.05 for all). RQ refers to relative quantity of mRNA level

Real-time quantitative PCR was performed to measure the expression levels of VEGF, CD31, vWF, b-FGF, NGF, and NT-3 mRNA. After SHED transplantation, the expression levels of CD31, vWF, b-FGF, NGF, and NT-3 mRNA were significantly higher than those in the saline group (*P* < 0.05, for all; Fig. 6).

### Western blot analysis

**Fig. 7:**
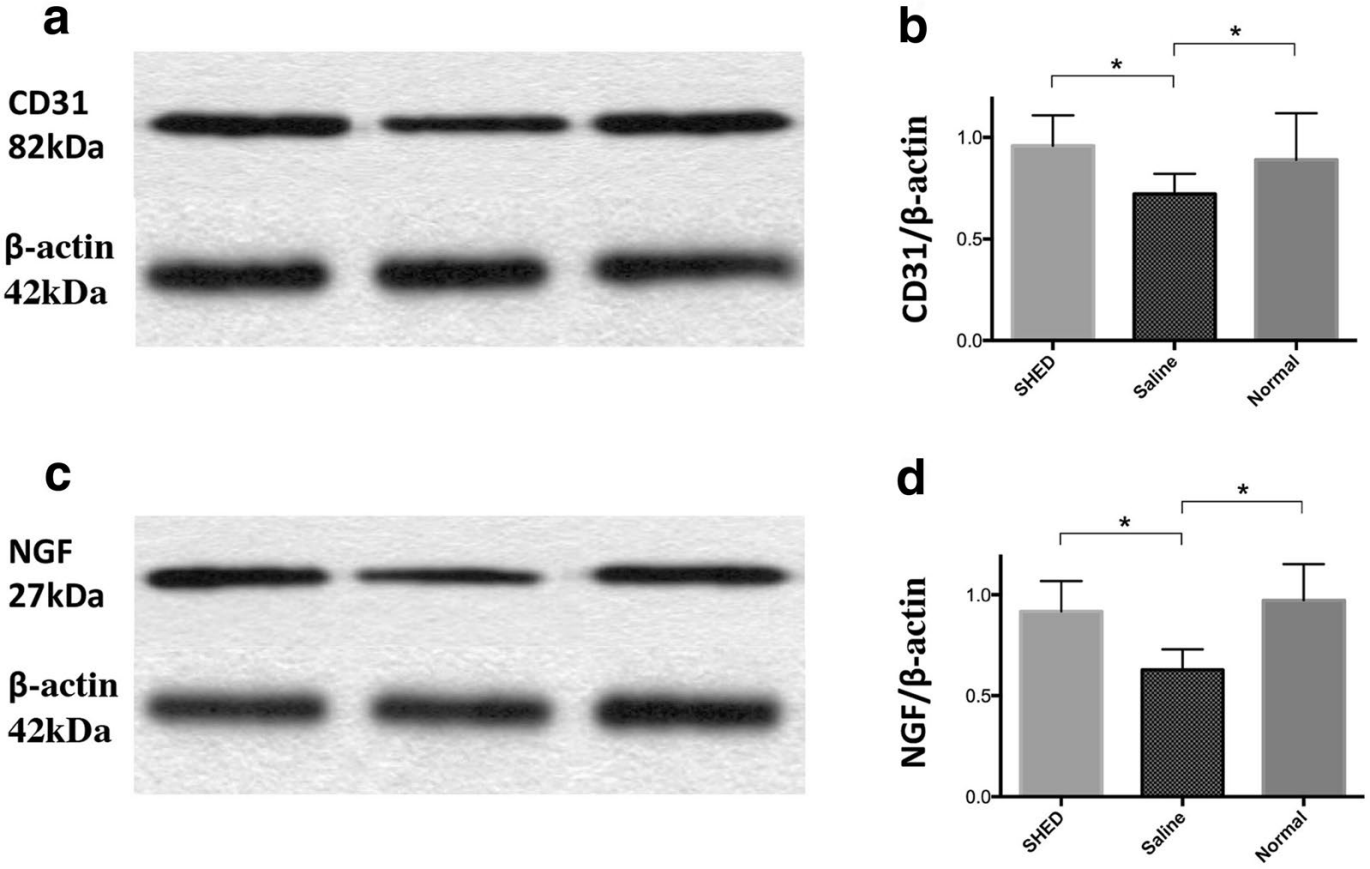
Western blot analysis. The grouping of blots were cropped from different fields. The protein levels of CD31 (**a**, **b**) and NGF (**c**, **d**) were higher in the SHED group in the saline group (*P < 0.05)

The protein levels of CD31 and NGF in the SHED group were significantly higher than those in the saline group (*P* < 0.05, for all; Fig. 7).

## Discussion

Stem cells from human exfoliated deciduous teeth (SHED) have been isolated from naturally exfoliated deciduous teeth with the capacity to differentiate into osteogenic and odontogenic cells, adipocytes, and neural cells. SHEDs are derived from a very accessible tissue resource and are capable of providing enough cells for potential clinical application via their high proliferation rate and expression of telomerase [17]. In addition, SHED have immunomodulatory abilities [18, 20]. SHED have a good prospect in the treatment of T1DM, liver fibrosis, lupus erythematosus, and spinal cord injury. The safety of SHED has been verified by our previous study [21], moreover, a clinical study related SHED transplantation in human indicates SHED is safe for humans during a 2 years observation [22].

Goto-Kakizaki (GK) rats have been widely used as a reliable animal model for Type 2 diabetes. Low insulin secretion and modest hyperglycemia are characteristic features of GK rats [23, 24]. It has been shown that these rats exhibit early thickening of the basement membrane of renal glomeruli [25] and reduction of motor nerve conduction velocity (MNCV) [26, 27]. Wada R’s study [28] suggested GK rats showed fasting hyperglycemia after 8 weeks of age, and slowing of MNCV to 80% of normal control levels was detected in GK rats. Teased fiber studies revealed a higher incidence of fibers with paranodal, segmental demyelination and axonal degeneration in GK rats. Based on the above reports, GK rats were chosen for establishing the animal model by feeding a high-fat diet in this study.

The present study demonstrated that SHED transplantation into the caudal vein of diabetic GK rats SHED transplantation increased the density of small vessels and improved nerve function, thereby alleviating the persistent neuropathic pain (mechanical hyperalgesia) they experienced.

VFH is a widely used noninvasive method to assess tactile pain. To calculate the threshold value, in the present study, we used the classic Dixon’s up-and-down method [19], and more accurate results from behavioral experiments were obtained using multiple stimulations. With observation time and the increase in blood sugar concentration, diabetic rats became more sensitive to mechanical pain in weeks 1–5. PWMT gradually decreases with time, which means the sensitivity of the diabetic rats for mechanical stimulation increases gradually with the development of diabetes. After the intervention, the PWMT of GK rats in the SHED group increased, and statistical analysis showed that the PWMT values of GK rats in the SHED group at weeks 7 and 8 were significantly higher than that in the saline group (*P *< 0.05). Thus, SHEDs can significantly reduce the sensitivity of diabetic rats to mechanical stimulation.

Damage to the peripheral blood vessels is one of the main factors affecting DPN, and the degree of microvascular lesions is associated with the severity of neuropathy. The main manifestations of the microvascular lesion are vascular endothelial cell proliferation, swelling, and the consequent thickening of the basement membrane of the vascular wall, which is mainly caused by the continuous high glucose status. These changes affect oxygen transport, causing nerve ischemia and hypoxia and leading to neural necrosis or apoptosis [29].

With the development of immunohistochemical techniques, fibers in the epidermis can be observed as physiological and pathological characteristics, which expressed the particular neural markers PGP9.5 positively. Among the many indicators that enable observation of epidermal nerve fibers, IENFD has been established as a useful research tool for unmyelinated C and thinly myelinated A delta fibers and is considered as a surrogate marker for small fiber neuropathy. The IENFD-based method is reliable for assessing functional damage in small fibers and has been widely used in clinical diagnosis [30]. In the present study, we found that the capillary to muscle fiber ratio and IENFD in GK rats were significantly lower than those in normal rats, but SHED transplantation can increase the levels of these indicators in the experimental rats to ones higher than those in the saline group. The pathological effects of diabetes on nerve fibers are axonal degeneration and segmental demyelination. In patients with chronic diabetes, the number of axons is decreased, and the distal skeletal muscles show neurogenic atrophy. The neuropathological results of the present study showed that the sciatic nerve pathology in the SHED group was better than that in the saline group. They indicate that SHED can improve blood flow in large and small fiber nerves and muscles.

Various growth factors are considered to play an important role in the pathogenesis of DPN, of which factors related to microcirculation and nerve growth and nutrition are the most important. Clinical vasodilator therapy has a particular effect on DPN. CD31, VEGF, and vWF participate in the formation of vascular endothelial cells and blood vessels [31, 32]. The biological effects of bFGF are also extensive. It has the functions of promoting blood vessel formation, wound healing, and tissue repair and plays an important role in nerve growth and tissue regeneration. NGF is an essential factor in the growth, development, and functional maintenance of sensory, sympathetic, and central cholinergic neurons. Synthesis of NGF is reduced in diabetes because of insulin deficiency and Schwann cell damage [33], whereas a decrease in NGF can result in axoplasmic transport and negative effects on NGF receptor expression, which affects the regulation of related gene expression and ultimately neurotrophy. NT-3 has also been confirmed to play an important role in peripheral nerve repair. Implantation of a fibronectin mesh impregnated with NT-3 solution into a rat sciatic nerve 10-mm-injury model resulted in a significant increase in the number of myelinated axons. Studies also confirmed that NT-3 can improve the sensory nerve function in diabetic rats, demonstrating that a reduction in NT-3 plays an important role in the development of DPN [33]. Previous studies have shown that SHEDs have the potential for endothelial and neural differentiation. At the mRNA level, the expressions of CD31, vWF, bFGF, NGF, and NT3 in the tissues of the SHED group were higher than those in the tissues of the saline group. The expression of CD31 and NGF at the protein level was also higher in the SHED group than that in the saline group, and no significant differences were found between the normal and SHED groups. Studies have proven that SHEDs can induce the formation of new blood vessels, promote blood vessel growth, relieve peripheral microcirculation disorders, generate neurotrophic factors, and slow down the neuropathological changes related to diabetes, thereby preventing the occurrence of DPN and slowing its progress.

In the present study, SHEDs and saline were injected into the caudal veins of GK and normal control rats. Both local injection and the intravenous infusion method have been used in stem cell-based treatment of diabetes complications. Since local injection increases the local concentration and number of stem cells, it may not be useful for DPN, which does not involve just local trauma but also involves systemic hyperglycemia, circulatory disorders, and oxidative stress. In contrast, the intravenous infusion method is more consistent with the principle of stem cell homing and chemotaxis. In the present study, GFP-SHEDs were found to localize in the muscle tissue, indicating that intravenously injected SHEDs can colonize local tissues. However, the optimal number of cells and injections for stem cell therapy differ among studies. Future work should compare intravenous injection, local injection, or a combination in order to identify the best treatment protocol. Further, individualized treatment plans should be developed in order to achieve the optimal therapeutic effects.

## Conclusion

In summary, SHED transplantation can prevent the development of DPN by participating in tissue regeneration, increasing local blood flow, and conferring neurotrophic protection. Local cell differentiation and paracrine function may be the mechanisms underlying SHED-based tissue repair. Stem cell therapy involving SHEDs and the mechanism of its therapeutic effects need to be further examined.

## Data Availability

This manuscript has not been published in whole or in part nor is it being considered for publication elsewhere.

## Abbreviations

IENFD: intra-epidermal nerve fiber density
GFP: green fluorescence protein
GK: Goto-Kakizaki
DPN: diabetic peripheral neuropathy
SHED: stem cells from human exfoliated deciduous teeth
PWMT: paw withdrawal mechanical threshold
VFH: von Frey hairs
vWF: von Willebrand factor
bFGF: basic fibroblast growth factor
VEGF: vascular endothelial growth factor
NGF: nerve growth factor
NT-3: neurotrophin-3
GAPDH: glyceraldehyde-phosphate dehydrogenase

## References

[1] Ogurtsova K, da Rocha Fernandes JD, Huang Y, Linnenkamp U, Guariguata L, Cho NH, Cavan D, Shaw JE, Makaroff LE (2017). IDF diabetes atlas: global estimates for the prevalence of diabetes for 2015 and 2040. Diabetes Res Clin Pract.

[2] England JD, Gronseth GS, Franklin G, Miller RG, Asbury AK, Carter GT, Cohen JA, Fisher MA, Howard JF, Kinsella LJ, Latov N, Lewis RA, Low PA, Sumner AJ, American Academy of N, American Association of Electrodiagnostic M, American Academy of Physical M, Rehabilitation (2005). Distal symmetric polyneuropathy: a definition for clinical research: report of the American Academy of Neurology, the American Association of Electrodiagnostic Medicine, and the American Academy of Physical Medicine and Rehabilitation. Neurology.

[3] Gibbons GW, Shaw PM (2012). Diabetic vascular disease: characteristics of vascular disease unique to the diabetic patient. Semin Vasc Surg.

[4] Shun CT, Chang YC, Wu HP, Hsieh SC, Lin WM, Lin YH, Tai TY, Hsieh ST (2004). Skin denervation in type 2 diabetes: correlations with diabetic duration and functional impairments. Brain.

[5] Forbes JM, Cooper ME (2013). Mechanisms of diabetic complications. Physiol Rev.

[6] Duran-Jimenez B, Dobler D, Moffatt S, Rabbani N, Streuli CH, Thornalley PJ, Tomlinson DR, Gardiner NJ (2009). Advanced glycation end products in extracellular matrix proteins contribute to the failure of sensory nerve regeneration in diabetes. Diabetes.

[7] Liu GS, Shi JY, Lai CL, Hong YR, Shin SJ, Huang HT, Lam HC, Wen ZH, Hsu KS, Chen CH, Howng SL, Tai MH (2009). Peripheral gene transfer of glial cell-derived neurotrophic factor ameliorates neuropathic deficits in diabetic rats. Hum Gene Ther.

[8] Argoff CE, Backonja MM, Belgrade MJ, Bennett GJ, Clark MR, Cole BE, Fishbain DA, Irving GA, McCarberg BH, McLean MJ (2006). Consensus guidelines: treatment planning and options. Diabetic peripheral neuropathic pain. Mayo Clin Proc.

[9] Matlhagela K, Taub M (2006). Involvement of EP1 and EP2 receptors in the regulation of the Na, K-ATPase by prostaglandins in MDCK cells. Prostaglandins Other Lipid Mediat.

[10] Calcutt NA, Jolivalt CG, Fernyhough P (2008). Growth factors as therapeutics for diabetic neuropathy. Curr Drug Targets.

[11] Naruse K, Hamada Y, Nakashima E, Kato K, Mizubayashi R, Kamiya H, Yuzawa Y, Matsuo S, Murohara T, Matsubara T, Oiso Y, Nakamura J (2005). Therapeutic neovascularization using cord blood-derived endothelial progenitor cells for diabetic neuropathy. Diabetes.

[12] Okawa T, Kamiya H, Himeno T, Kato J, Seino Y, Fujiya A, Kondo M, Tsunekawa S, Naruse K, Hamada Y, Ozaki N, Cheng Z, Kito T, Suzuki H, Ito S, Oiso Y, Nakamura J, Isobe K (2013). Transplantation of neural crest-like cells derived from induced pluripotent stem cells improves diabetic polyneuropathy in mice. Cell Transplant.

[13] Kim H, Park JS, Choi YJ, Kim MO, Huh YH, Kim SW, Han JW, Lee J, Kim S, Houge MA, Ii M, Yoon YS (2009). Bone marrow mononuclear cells have neurovascular tropism and improve diabetic neuropathy. Stem Cells.

[14] Shibata T, Naruse K, Kamiya H, Kozakae M, Kondo M, Yasuda Y, Nakamura N, Ota K, Tosaki T, Matsuki T, Nakashima E, Hamada Y, Oiso Y, Nakamura J (2008). Transplantation of bone marrow-derived mesenchymal stem cells improves diabetic polyneuropathy in rats. Diabetes.

[15] Kim BJ, Jin HK, Bae JS (2011). Bone marrow-derived mesenchymal stem cells improve the functioning of neurotrophic factors in a mouse model of diabetic neuropathy. Lab Anim Res.

[16] Hata M, Omi M, Kobayashi Y, Nakamura N, Tosaki T, Miyabe M, Kojima N, Kubo K, Ozawa S, Maeda H, Tanaka Y, Matsubara T, Naruse K (2015). Transplantation of cultured dental pulp stem cells into the skeletal muscles ameliorated diabetic polyneuropathy: therapeutic plausibility of freshly isolated and cryopreserved dental pulp stem cells. Stem Cell Res Ther.

[17] Miura M, Gronthos S, Zhao M, Lu B, Fisher LW, Robey PG, Shi S (2003). SHED: stem cells from human exfoliated deciduous teeth. Proc Natl Acad Sci U S A.

[18] Yamaza T, Kentaro A, Chen C, Liu Y, Shi Y, Gronthos S, Wang S, Shi S (2010). Immunomodulatory properties of stem cells from human exfoliated deciduous teeth. Stem Cell Res Ther.

[19] Dixon WJ (1980). Efficient analysis of experimental observations. Annu Rev Pharmacol Toxicol.

[20] Dai Yu-Yang, Ni Si-Yang, Ma Ke, Ma Yu-Shi, Wang Zhi-Shi, Zhao Xiu-Li (2019). Stem cells from human exfoliated deciduous teeth correct the immune imbalance of allergic rhinitis via Treg cells in vivo and in vitro. Stem Cell Res Ther.

[21] Li J, Xu SQ, Zhao YM, Yu S, Ge LH, Xu BH (2018). Comparison of the biological characteristics of human mesenchymal stem cells derived from exfoliated deciduous teeth, bone marrow, gingival tissue, and umbilical cord. Mol Med Rep.

[22] Xuan K, Li B, Guo H, Sun W, Kou X, He X, Zhang Y, Sun J, Liu A, Liao L, Liu S, Liu W, Hu C, Shi S, Jin Y (2018). Deciduous autologous tooth stem cells regenerate dental pulp after implantation into injured teeth. Sci Transl Med.

[23] Goto Y, Kakizaki M, Masaki N (1975). Spontaneous diabetes produced by selective breeding of normal Wistar rats. Proc Jpn Acad.

[24] Portha B, Serradas P, Bailbe D, Suzuki KI, Goto Y, Giroix MH (1991). β-Cell insensitivity to glucose in the GK Rat, a spontaneous nonobese model for type II diabetes. Diabetes.

[25] Yagihashi S, Goto Y, Kakizaki M, Kaseda N (1978). Thickening of glomerular basement membrane in spontaneously diabetic rats. Diabetologia.

[26] Yagihashi S, Tonosaki A, Yamada KI, Kakizaki M (1982). Peripheral neuropathy in selectively-inbred spontaneously diabetic rats: electrophysiological, morphometrical and freeze-replica studies. Tohoku J Exp Med.

[27] Suzuki K, Yenchung H, Toyota T, Goto Y, Hirata Y (1990). The significance of nerve sugar levels for the peripheral nerve impairment of spontaneously diabetic GK (Goto-Kakizaki) rats. Diabetes Res.

[28] Wada R, Koyama M, Mizukami H, Odaka H, Ikeda H, Yagihashi S (1999). Effects of long-term treatment with α-glucosidase inhibitor on the peripheral nerve function and structure in Goto-Kakizaki rats: a genetic model for type 2 diabetes. Diabetes Metab Res Rev.

[29] Yagihashi S (2002). Pathology of diabetic neuropathy; a review from the updated literature of the last 10 years. Nihon Rinsho.

[30] Tesfaye S, Boulton AJ, Dyck PJ, Freeman R, Horowitz M, Kempler P, Lauria G, Malik RA, Spallone V, Vinik A, Bernardi L, Valensi P, Toronto Diabetic Neuropathy Expert G (2010). Diabetic neuropathies: update on definitions, diagnostic criteria, estimation of severity, and treatments. Diabetes Care.

[31] Park S, Sorenson CM, Sheibani N (2015). PECAM-1 isoforms, eNOS and endoglin axis in regulation of angiogenesis. Clin Sci (Lond).

[32] Rondaij MG, Bierings R, Kragt A, van Mourik JA, Voorberg J (2006). Dynamics and plasticity of Weibel-Palade bodies in endothelial cells. Arterioscler Thromb Vasc Biol.

[33] Unger JW, Klitzsch T, Pera S, Reiter R (1998). Nerve growth factor (NGF) and diabetic neuropathy in the rat: morphological investigations of the sural nerve, dorsal root ganglion, and spinal cord. Exp Neurol.

